# Local Anesthesia in Dental Surgery1Abstract of a paper read before the Massachusetts Dental Society, May 2-4 1912, Boston, Mass. and published in the Sept. 1912, Journal of Allied Societies

**Published:** 1912-11-15

**Authors:** Tandlaege Imm Ottesen

**Affiliations:** Christiania, Norway


### LOCAL ANESTHESIA IN DENTAL SURGERY[1]

BY HERR TANDLAEGE IMM OTTESEN, CHRISTIANIA, NORWAY.

During the last seven years I have used exclusively local anesthetics in all operations of dental surgery, such as extractions, amputation of roots, extraction of pulp, opening of the antrum, preparation of cavities, and separation of teeth. As long as we had to use exclusively cocain and before the discovery of adrenalin, local anesthesia could not be so generally employed because cocain alone was too toxic and the anesthesia did not last long enough to permit of finishing the operation. Later on, after Einhorn had produced novocain, and after the discovery of the artery-contracting effect of adrenalin, local anesthesia by injection came to be more generally employed. We are deeply indebted to Dr. Braun for his excellent research work in studying the pharmo-dynamic influence of different anesthetics.

1Abstract of a paper read before the Massachusetts Dental Society, May 2-4 1912, Boston, Mass. and published in the Sept. 1912, JOURNAL OF ALLIED SOCIETIES

He found that the state of anesthesia is due to a chemical combination between the elements of the cells and the anesthetic and not, as Dr. Schleich maintained, to the pressure of the solution on the nerve-fibers. Dr. Braun also pointed out what great importance arterial contraction at the point of injection has for the effectiveness and duration of the anesthesia. Adrenalin, and, still better, synthetic suprarenin are indispensable additions to every anesthetic solution.

The first place among the local anesthetics of the present day, I believe is held by novocain. Cocain has a greater anesthetic effect, but it is too toxic and cannot be sterilized by boiling. Novocain fulfills the chief requirements which Braun demands of an anesthetic. 1. It is as little toxic as possible in proportion to its anesthetic power. 2. It causes no changes in the tissues. 3. It can be used in combination with adrenalin and suprarenin. 4. It is easily soluble in water and can be boiled. Novovain is a white powder, and forms together with water a neutral solution which has the same effect on the sensitive nerves as cocain. A 1 ½percent. solution is sufficient to anesthetize so large a nerve as the nervus ischiadicus. General effects after absorption are hardly ever noticeable. If novocain is applied to an inflamed wound you will rather find a soothing effect than an irritating. All who have used novocain are agreed that it is a relatively non-toxic substitute for cocain, and that it is indispensable in dental practice when painless treatment is demanded.

We employ it in a solution of from 1 to 4 per cent. strength, together with one drop of adrenalin or suprarenin to each cubic centimeter of solution. It is advisable to add small quantities of thymol and sodium chloride. I myself at present use a solution recommended by Fischer—viz.:

| | |
|---|---|
| Thymol | ½ grain. |
| Sodium chloride | 14 grains. |
| Distilled water | 3½ ounces. |

Of this solution I take 100 drops and add to it, as a rule, 2 grains of novocain, which makes a 2 per cent. solution; boil it and add one drop of adrenalin or suprarenin, and draw it into the sterilized syringe. This novocain solution I make up freshly every day.

To make the injections properly it is necessary to be thoroughly acquainted with the anatomy of the jaws and teeth. In examining a skull of the labial and buccal portions of the upper and lower jaws, it will be noted that there are several small tubular perforations above the apex of the roots of the front teeth and in the septum between the teeth as well as in the region of the bicuspids in the upper jaw. In the lower jaw you will notice that these perforations are not so numerous. In the fossa mentalis will be found some small canals. In some cases you will see that these perforations are not at all numerous. This is due to the age and individual differences, and that is the reason why in some cases you will have perfect anesthesia with gingival injection, and in some cases you will not. These perforations are more numerous in young persons than in old.

On the lingual side in the upper jaw are many perforations, through which the novocain solution, by gingival injection, can penetrate into the spongiosa. Far back in the region of the lingual root of the wisdom tooth is situated the foramen palatinum anterior. By injection here and on the tuber maxillare, you will obtain an excellent result by means of "conduction anesthesia." The anesthesia will reach as far as the wisdom tooth, the second molar, and the first molar.

On the lingual side of the lower jaw you will not find many perforations, but there are some in the region of the front teeth.

The foramen mandibulare is an excellent point for injection to obtain conduction anesthesia for the lower molars. This foramen is found by moving the finger along the linea obliqua externa to the lingual side of the ramus about one centimeter above the occlusal surface of the wisdom tooth. Another very important foramen for conduction anesthesia is the foramen infra orbitale, a foramen easily found underneath the lower orbital ridge. An injection here will anesthetize the region of the front teeth and also the cuspid and the bicuspids. The foramen mentale on the lower jaw is not so often used in the technique of injection. In some cases, for anesthetizing the bicuspids, an injection here will give good results.

We can divide the technics of injection into three groups —namely, 1. *Gingival injection.* 2. "*Leitungsanesthesie*" (con-conduction anesthesia). 3. *Intra-alveolar injection.* By the first named method you will, as a rule, obtain good results with nearly all the teeth of the upper jaw. The cuspids will sometimes be found difficult to anesthetize by this method, because, as you will remember, there are few openings in the bone in that region, so that the solution cannot easily penetrate into the spongiosa.

Before we commence making any injection we must make sure of the patient's state of health. If the patient has any heart weakness so that he is liable to be affected by the novocain-adrenalin, it is advisable to use a weaker solution, 1 or $1\frac{1}{2}$ per cent., and to employ a compression bandage round the neck, as advised by Professor Guido Fischer. This bandage is to be made so tight that the patient's face becomes a little flushed. Before making the injection I always sterilize the gum by carefully rubbing it with a cotton pellet dipped in a solution of tincture of iodine and tincture of aconite in equal parts. If you have a very sensitive patient, so that you want to make the puncture with the injection-needle quite painless you can spray the gum a little with ethyl chloride.

In obtaining anesthesia of the front teeth the puncture is made through the gingiva and underneath the periosteum, and it is very important to have the flat part of the needle-point pressed against the bone. Now inject the solution very slowly and gently with a steadily increasing pressure, and at the same time massage the gum a little with the left forefinger. Twenty drops of the solution will be enough in most cases to obtain anesthesia in the central and lateral incisors about five minutes after injection. The anesthesia will last from twenty to thirty minutes and sometimes longer.

The point of injection foı the bicuspids is directly over the root apex. The injection is performed in the same manner as with front teeth. As a rule, it is enough to inject only on the buccal or labial side, but there is no objection to also injecting a few drops on the lingual side, making the puncture in the same manner as described above. The fewer punctures the better. When employing gingival injection for the molars it will generally be necessary to inject also on the lingual side in the region of the apex of the roots. But in the case of these teeth you will get better results by using intra-alveolar injections or conduction anesthesia, as I will describe later on.

In the lower jaw it will be very difficult to obtain good results by gingival injection. As far as the front teeth are concerned, the result may be satisfactory enough by the canula being directed to the region of the fossa mentalis, where the bone is porous. One cubic centimeter is enough to inject. When employing gingival injection for the bicuspids and molars you had better inject, with cosiderable pressure, in the inter-dental papilla, because the septum of the bone between those teeth is porous, but it is not advisable to employ this method, because, due to the high pressure, you may cause inflammation or necrosis in the gingival. Here there is indication for intra-alveolar injection or "Leitungsanesthesia." I never employ gingival or intro-ossal injections if there is inflammation.

Conduction anesthesia ("Leitungsanesthesia,") as advised by Guido Fischer, offers great advantages in extracting teeth affected with periostitis, or in the surgical treatment of abscess.

In mandibular anesthesia, the syringe is placed opposite the bicuspids on the lingual side, and the needle is forced through the mucous membrane into the region of the foramen mandibulare. Then inject fifteen drops, using massage and after fifteen or twenty minutes you will obtain anesthesia from the wisdom tooth to the second bicuspid. I use a long needle and a syringe designed by Professor

Williger. In mandibular anesthesia you must be prepared for failure occasionally, until you have experience in finding the right place for injection.

Another good place for obtaining conduction anesthesia for the upper front teeth, cuspids and bicuspids is the foramen infra-orbitale. With the left thumb you can feel the lower orbital ridge, under which in the center you have the infra-orbital foramen. With the left fore-finger you then raise the lip and force the long needle through the tissue up to the foramen, and slowly inject fifteen drops, employing massage during and after injection. A more perfect anesthesia can be obtained by also injecting on the lingual side of the teeth you wish to anesthetize. There is indication for this form of injection in surgical treatment of abscesses beyond the apex of the teeth from the medial to the bicuspid tooth. Anesthesia will occur about ten or fifteen minutes after injection and will last thirty minutes and longer, so that you will have time enough to complete the operation. To obtain conduction anesthesia of the upper molars, you may inject fifteen drops on the tuber maxillare and ten drops in the region of the foramen *palatinale* anterior. It is convenient to use a double-angled attachment to the syringe with a needle 17.5 millimeter long. With this attachment you can easily force the canula upward and backward to the tuberosity, where we have the foramen through which the nervus alveolaris superior posterior passes into the maxilla, providing sensitive nerves for the upper molars. There must also, as mentioned before, be injected a half or one cubic centimeter at the foramen palatinale anterior, which is easily found on the lingual side in the region of the wisdom tooth.

It is advisable to support the effect of the anesthesia for the first molar by also injecting one and a half cubic centimeter in the region of the apex of the lingual root. This method of injection will seldom fail, and is especially suitable in extracting molars affected with periostitis, because you avoid injecting into the inflamed parts.

The third method of injecting, the intra-alveolar, offers great advantages over the two methods described—namely: 1. The effect of the injection is more certain. 2. The quantity of the solution can be reduced. 3. Anesthesia is obtained immediately after injection. This method was first used and described by Dr. Joseph Otte, of Holland, in a paper read before the Nederlanske Tandmeesteer-Vereeinging in 1896. He describes the method as very practicable in extraction with the use of cocain solution without adrenalin, which had not then been discovered. Otte made the perforation into the spongiosa at nearly right angles to the root, using a trepan-drill one millimeter in diameter, and after having first obtained anesthesia of the soft part of the gums. This method was not very widely employed until later on, when the method had been improved and developed in Norway, and even at the present time it is not very generally adopted outside Norway. Four years ago I gave a demonstration of this method to a private gathering of dentists in Chicago. They admired the excellent results obtained by the method, but nevertheless were inclined to be skeptical, as they feared the danger of infection which might give rise to serious forms of periostitis. However, statistics in Norway extending over several thousand cases prove that if the injection is made with scrupulous care and aseptically from beginning to end, there will be no risk of infection, while you will have a certain method of securing painless treatment.

The Norwegian improved method is essentially the same as Otte's, except that the perforation is made on a level with the neck of the tooth and parallel to the long axis. Instead a trepan-drill we use a Beutelrock root-canal drill of the same diamater as the canula of the syringe.

When employing intra-alveolar injection you have to take the same precautions as in the two other methods. First and last, work *aseptically*. The syringe needle, drill, and solution must be absolutely sterilized, and this is best secured by boiling. Then rub the gum thoroughly with the iodine-aconite solution, and keep the place for injection free from the flow of saliva. Then inject a little into the gingiva, about two millimeters from the tip of the interdental papilla, wait one or two minutes and then bore through the hard outer part of the bone into the spongiosa. Great care must be taken to reach the septum between the roots without touching the pericementum. Otherwise, there will be soreness of the tooth lasting for several days. Therefore, in carrying out the injection it is necessary to be perfectly acquainted with the position of the roots in the jaw.

For the lower jaw it will be very convenient to use a right angled attachment to the syringe, and of course also a right angled hand piece to the engine to penetrate the bone when anesthetizing the first and second molars. As a rule, the perforation is nearly always made on the buccal side, but if the crowns of the teeth are inclined outward it is more convenient to operate on the lingual side. To be sure of good results you can go as far as to the end of the roots. By injecting here you will nearly always secure anesthesia of the first and second molars, but it is always a good practice to inject on the distal side of the tooth you wish to anesthetize.

By injecting on the mesial side of the cuspids you will nearly always obtain anesthesia of both the lateral and medial lower incisors. When anesthetizing the upper wisdom tooth it will sometimes be difficult to hit upon the exact spot for injection into the bone, and in this case it will be best to employ gingival injection or conduction anesthesia. When using intra-alveolar injection for the first and second molars I prefer, especially if the cheek is thick and stiff, to penetrate on the lingual side, directing the drill between the lingual roots in an upward and outward direction. As regards the bicuspids, it is easy to operate on the buccal side, either between the first molar and the second bicuspid, or between the bicuspids.

The upper cuspid tooth is sometimes a little difficult to anesthetize, but if you penetrate a little more than half the root length, you will always secure good results. The upper front teeth are, as said before, easy to anesthetize by gingival injection, but it is also quite practicable to use intra-alveolar injection. When penetrating between the lateral and central teeth, care must be taken to avoid touching the pericementum, because as you know the roots of these teeth do not diverge much, so that the space between them is narrow. You will notice that the space between the centrals is sufficient to operate upon without any risk of touch ing the pericementum with the drill.

Before pushing the syringe needle into the spongiosa you must carefully sterilize it, either by drawing it through the flame of a spirit lamp, or, still better, by leaving it in boiling water between the first and second injections. Then take care that there are no air-bubbles in the syringe, and inject the solution with a very gentle pressure. About a quarter of a cubic centimeter is sufficient to produce anesthesia. Maintain the pressure while withdrawing the canula, so that the passage becomes filled with solution, thereby excluding a possible risk of infection through the hole. Such risk, however, has been proved to be practically non-existent. We have fully discussed this point in our dental societies in Norway, and we have come to the conclusion that the contraction of the tissues follows so closely on the withdrawal of the needle as to preclude the possibility of an invasion of microbes.

In concluding this paper I want to emphasize the most important points to be considered when employing local anesthesia. In the first place, the general health of the patient must be taken into account. If any heart disease or other weakness is present, employ, as already mentioned a compression bandage around the neck and use a weaker solution. Secondly, be careful to select the method of injection most suitable to the case in hand. Above all, *work aseptically at every step of the operation.* Keep the patient under observation during and after injection.—*Journal of Allied Societies.*

